# Prominent astrocytic alpha-synuclein pathology with unique post-translational modification signatures unveiled across Lewy body disorders

**DOI:** 10.1186/s40478-022-01468-8

**Published:** 2022-11-12

**Authors:** Melek Firat Altay, Alan King Lun Liu, Janice L. Holton, Laura Parkkinen, Hilal A. Lashuel

**Affiliations:** 1 Laboratory of Molecular and Chemical Biology of Neurodegeneration, Brain Mind Institute, Ecole Polytechnique Fédérale de Lausanne, EPFL, 1015 Lausanne, Switzerland; 2 Oxford Parkinson’s Disease Centre, University of Oxford, Oxford, UK; 3 Nuffield Department of Clinical Neurosciences, John Radcliffe Hospital, University of Oxford, West Wing, Level 6, Oxford, OX3 9DU UK; 4 Queen Square Brain Bank for Neurological Disorders, University College London Queen Square Institute of Neurology, London, England

**Keywords:** Alpha-synuclein, Parkinson’s disease, Lewy body disorder, Aggregation, Post-translational modification, Astrocytes

## Abstract

Alpha-synuclein (aSyn) is a pre-synaptic monomeric protein that can form aggregates in neurons in Parkinson’s disease (PD), Parkinson’s disease with dementia (PDD) and dementia with Lewy bodies (DLB), and in oligodendrocytes in multiple system atrophy (MSA). Although aSyn in astrocytes has previously been described in PD, PDD and DLB, the biochemical properties and topographical distribution of astrocytic aSyn have not been studied in detail. Here, we present a systematic investigation of aSyn astrocytic pathology using an expanded antibody toolset covering the entire sequence and key post-translational modifications (PTMs) of aSyn in Lewy body disorders (LBDs) and in MSA. Astrocytic aSyn was detected in the limbic cortical regions of LBDs but were absent in main pathological regions of MSA. The astrocytic aSyn was revealed only with antibodies against the mid N-terminal and non-amyloid component (NAC) regions covering aSyn residues 34–99. The astroglial accumulations were negative to canonical aSyn aggregation markers, including p62, ubiquitin and aSyn pS129, but positive for phosphorylated and nitrated forms of aSyn at Tyrosine 39 (Y39), and not resistant to proteinase K. Our findings suggest that astrocytic aSyn accumulations represent a major part of aSyn pathology in LBDs and possess a distinct sequence and PTM signature that is characterized by both N- and C-terminal truncations and modifications at Y39. This is the first description that aSyn accumulations are made solely from N- and C-terminally cleaved aSyn species and the first report demonstrating that astrocytic aSyn is a mixture of Y39 phosphorylated and nitrated species. These observations underscore the importance of systematic characterization of aSyn accumulations in different cell types to capture the aSyn pathological diversity in the brain. Our findings combined with further studies on the role of astrocytic pathology in the progression of LBDs can pave the way towards identifying novel disease mechanisms and therapeutic targets.

**Supplementary Information:**

The online version contains supplementary material available at 10.1186/s40478-022-01468-8.

## Introduction

Parkinson’s disease (PD), Parkinson’s disease with dementia (PDD), dementia with Lewy bodies (DLB), and multiple system atrophy (MSA) are age-related neurodegenerative diseases that are characterized by the abnormal accumulation and aggregation of alpha-synuclein (aSyn). Hence, they are collectively referred to as synucleinopathies. PD, PDD and DLB typically involve the accumulation of aSyn in the neuronal soma and processes as Lewy bodies (LBs) and Lewy neurites (LNs), respectively [1, 2]. MSA, on the other hand, is characterized by the inclusion formation mainly in the oligodendrocytes called glial cytoplasmic inclusions (GCIs) [3].

In the early 2000s, several studies reported the presence of aSyn-positive astrocytes in LB diseases [4–8] (Table 1). Yet, despite the increasing number of publications aimed at dissecting the molecular and structural features of aSyn pathology, very little is known about the biochemical properties and distribution of aSyn species associated with the astrocytes, how they form, and what role they may have in the pathogenesis of PD and other synucleinopathies. Only a small number of studies (13) have been published, using a limited number of antibodies, on the characterization of aSyn astrocytic pathology and its relationship to aSyn neuronal pathology, disease stage or duration [4–16].

**Table 1:** The studies since 2000 investigating the astrocytic aSyn pathology in human disease brains

Study	aSyn antibody	aSyn epitope	Disorder	Anatomical regions detected	Characteristics
Shoji et al. [5]	MDV	1–15	DLBD	Cerebral cortex, basal ganglia	Star-like
			PD	Cerebral cortex	Dot-like
Takeda et al. [6]	anti-NAC	61–75	LBD	Neocortex, hippocampus, brainstem	Star-like negative for ubiquitin
Terada et al. [16]	Chemicon	Unknown	DLB	Temporal, insular, cingulate and fronto-orbital cortices, amygdala, hippocampus	Star-like positive with GB and GFAP double staining negative for aSyn, ubiquitin or tau
Wakabayashi et al. [8]	NACP-1	Unknown	PD	Midbrain	Circular or coil-like, argyrophilic positive with GB staining which of the aSyn antibodies detect astrocytic aSyn not specified
	NACP-3	121–140			
	NACP-5	114–131			
Hishikawa et al. [4]	FL140	61–95	PD	Striatum, substantia nigra, pons, medulla, cerebellum, spinal cord	Circular or coil-like, argyrophilic distribution of glial accumulations not exclusive to astrocytic aSyn - refers to astrocytic and oligodendroglial aSyn distribution combined
Terada et al. [7]	EQV1	61–76	DLBD	Temporal, cingulate, fronto-orbital, parietal, insular cortices, amygdala, basal ganglia	Star-like, argyrophilic positive with GB staining not resistant to PK
Braak et al. [9]	SYN-1	91–99	PD	Amygdala, thalamus, septum, striatum, claustrum and cerebral cortex	Star-like negative for ubiquitin, p62 negative with modified Gallyas or Campbell-Switzer staining
Song et al. [14]	SYN-1	91–99	PD	Pons	Cytoplasmic staining
	Abcam	Unknown			
Kovacs et al. [12]	5G4	44–57//agg-aSyn	DLB	Amygdala, cingulate cortex	Star-like
Kovacs et al. [11]	5G4	44–57//agg-aSyn	PD, DLB	Entorhinal and temporal cortices, amygdala, striatum, hippocampus	Star-like (cortex and amygdala) granular cytoplasmic (striatum)
Nakamura et al. [13]	Wako	pS129	MSA	Medulla, pons, midbrain, spinal cord, lateral ventricles	Granular, non-fibrillar negative for ubiquitin, p62 negative with GB staining
	Abcam EP1536Y	pS129			
Fathy et al. [10]	SYN-1	91–99	ILBD, PD, PDD, DLB	Insula	Thin, fuzzy projections with some star-like accumulations; cytoplasmic aSyn in GFAP-positive astrocytes form a mesh-like structure
Sorrentino et al. [15]	3H11	43–63	DLB, AD/ALB	Amygdala	Star-like, granular
	5G4	44–57//agg-aSyn			

*AD/ALB*, Alzheimer's disease with amygdala restricted Lewy bodies; *aSyn*, alpha-synuclein; *agg-aSyn*, aggregated alpha-synuclein; *DLB*, dementia with Lewy bodies; *DLBD*, diffuse Lewy body disease; *GB*, Gallyas-Braak; *GFAP*, glial fibrillary acidic protein; *ILBD*, incidental Lewy body disease; *LBD*, Lewy body disease; *PD*, Parkinson's disease; *PDD*, Parkinson's disease with dementia; *PK*, proteinase K

One of the key reasons for the scarcity of data on astrocytic aSyn is that only a few of the aSyn antibodies appear to reveal these species. Although previous studies have reported that the astrocytic aSyn is detected by antibodies with epitopes against the NAC region of aSyn [9, 12, 15], they did not define the sequence properties of aSyn or identify the molecular determinants underpinning their observations. These astrocytic aSyn species are not revealed by the classical inclusion markers such as positivity for ubiquitin [6, 9, 11, 13, 16] and p62 [6, 9, 11, 13, 16], and their post-translational modification (PTM) profile and aggregation states have not been systematically investigated beyond two studies that assessed S129 phosphorylation (pS129) status [13, 15]. Furthermore, the antibodies used to characterise and diagnose aSyn pathology are often directed at the C-terminal domain of the protein, which could lead to the under-reporting of aSyn astrocytic pathology. The lack of appropriate tools and techniques that allow for the selective isolation and characterization of the astrocytic aSyn presents major challenges to defining their biochemical properties and relationship to other aSyn brain pathologies.

To address these challenges, we assessed aSyn astrocytic pathology using an expanded set of antibodies that target epitopes throughout the entire sequence of the protein and for the first time against 8 different aSyn PTMs. To further characterize the aggregation state of aSyn species in the astroglia, we used antibodies against known markers of LBs (ubiquitin, p62, and pS129) and aSyn aggregate-specific antibodies, and investigated their resistance to proteolysis by proteinase K. With the aim of shedding light on the properties of astrocytic aSyn pathology across different regions and synucleinopathies, we extended our studies to several limbic brain regions, including the entorhinal cortex, hippocampus, cingulate cortex, insula and amygdala of PD, PDD and DLB; and the pons, putamen, cerebellum, frontal cortex and occipital cortex of MSA.

Our studies show that astrocytic aSyn accumulations occur extensively in the limbic regions of LB disease cases. These aSyn species are post-translationally modified at Tyrosine 39 (Y39) and most likely cleaved at both the N- and C-termini of the protein, as evidenced by the fact that they are only detected with antibodies targeting epitopes approximately between residues 34–99. In addition to presenting new insight into the sequence and aggregation state of astrocytic aSyn, our work provides a validated toolset that should enable a more systematic re-assessment of the role of astrocytic aSyn pathology in the development and progression of synucleinopathies. Our findings also emphasise the importance of using an appropriate and validated detection tools capable of capturing the diversity of aSyn species to map and characterize aSyn pathology in astrocytes and other cell types.

## Materials and methods

### Antibodies

The primary and secondary antibodies used in this study are detailed in Additional file 1: Table S1.

### Human brain tissue samples

Post-mortem human brains stored at Queen Square Brain Bank (QSBB), Institute of Neurology in University College London (UCL), and Oxford Brain Bank (OBB), Nuffield Department of Clinical Neurosciences in University of Oxford, were collected in accordance with approved protocols by the London Multicentre Research Ethics Committee and the Ethics Committee of the University of Oxford (ref 15/SC/0639). All participants had given prior written informed consent for the brain donation. Both brain banks comply with the requirements of the Human Tissue Act 2004 and the Codes of Practice set by the Human Tissue Authority (HTA licence numbers 12198 for QSBB and 12217 for OBB). 3 cases of sporadic PD, MSA and familial PD with *SNCA* G51D mutation, 2 cases with PDD, 1 case with DLB and 1 case with PD with *SNCA* duplication were derived from QSBB, and 6 cases with sporadic PD, 4 cases with PDD, 2 cases with DLB and 3 healthy controls from OBB were used in this study. Case demographics are detailed in Table 2.

**Table 2:** Demographics of the human post-mortem cases included in this study. Positivity for astrocytic aSyn was assessed using the antibodies LASH-BL 34-45 or LASH-EGT nY39

Case ID	Gender (m/f)	Age at diagnosis (y)	Age at death (y)	Disease duration (y)	Postmortem delay (h)	Braak stage (LB-type)	Braak stage (AD-type)	Astrocytic aSyn detection		
								cing	EC	amg
PD1	m	66	78	12	32	6	4	+	+	+
PD2	m	73	82	9	55	6	1	+	−	+
PD3	m	87	92	5	36	6	1	+	+	+
PD4	m	57	80	23	na	5	1	+	+	+
PD5	m	73	79	6	72	5	2	+	−	−
PD6	m	63	69	6	72	5	2	+	+	+
PD7	m	55	75	20	88.5	6	1	+	−	−
PD8	m	80	89	8.5	26	6	4	−	na	na
PD9	m	60	84	24	71	6	2	+	na	na
PDD1	m	54	72	18	90	6	1	+	+	+
PDD2	m	71	80	9	64	6	3	+	+	+
PDD3	m	83	92	9	24	5	1	−	−	+
PDD4	m	77	91	14	72	6	2	+	−	+
PDD5	m	77	92	14	41	6	3	+	+	+
PDD6	m	61	80	19	66	6	2	+	na	na
DLB1	f	78	89	11.4	63	6	3	+	+	+
DLB2	m	62	72	10	48	6	1	na	+	na
DLB3	m	83	87	4	48	6	2	+	na	na
*SNCA* G51D1	m	19	49	30	43	na	na	+	+	na
*SNCA* G51D2	f	69	75	6.5	62	na	1	+	+	na
*SNCA* G51D3	m	45	52	6.5	85.3	na	1	+	+	+
*SNCA* duplication	m	55	62	6.9	143	6	2	+	+	+
MSA1	f	58	65	7.4	55	0	0	na	na	na
MSA2	m	67	74	6.6	22	0	3	na	na	na
MSA3	f	46	52	6	78	0	1	na	na	na
CTR1	f	na	89	na	24	0	2	−	−	−
CTR2	m	na	80	na	48	0	2	−	−	−
CTR3	f	na	89	na	24	0	2	−	−	−

*AD*, Alzheimer's disease; *amg*, amygdala; *aSyn*, alpha-synuclein; *cing*, cingulate cortex; *CTR*, control; *DLB*, dementia with Lewy bodies; *EC*, entorhinal cortex; *f*, female; *h*, hours; *m*, male; *LB*, Lewy body; *MSA*, multiple system atrophy; *na*, not assessed; *PD*, Parkinson's disease; *PDD*, Parkinson's disease with dementia; *SNCA*, synuclein-alpha; *y*, years

### Immunohistochemistry with 3,3′-diaminobenzidine (DAB) revelation and imaging

Formalin-fixed, paraffin-embedded (FFPE) sections were dewaxed in xylene and rehydrated through decreasing concentrations of industrial denatured alcohol (IDA). Antigen retrieval was carried out for the appropriate antibody (Additional file 1: Table S1). The rationale for the selection of the final antigen retrieval method and dilution for each antibody was so that the antibodies could reveal the most diverse/highest number of pathological inclusions. Autoclaving (AC) was run at 121 °C for 10 min in citrate buffer (pH 6.0). For formic acid (FA) pre-treatment, tissues were incubated in 80–100% FA for 15 min (except for 5 min with 5G4) at room temperature (RT). For PK pre-treatment, tissues were incubated at 37 °C for 5 min in 20 µg/mL of PK diluted in TE-CaCl_2_ buffer (50 mM Tris-base, 1 mM EDTA, 5 mM CaCl_2_, 0.5% Triton X-100, adjusted to pH8.0). Next, the sections were incubated for 30 min in 3% hydrogen peroxide in phosphate buffered saline (PBS) for quenching the endogenous peroxidase activity. Sections were briefly rinsed in distilled water and PBS, blocked in 10% foetal bovine serum (FBS) for 30 min at RT, and left at 4 °C overnight for incubation with the primary antibodies. Subsequently, the sections were washed in PBS-Tween 0.1% (PBS-T; 3 × 5 min) and incubated in the secondary antibody-horseradish peroxidase (HRP) complex as part of REAL EnVision detection system (Dako #K5007) for 1 h at RT. Sections were rinsed in PBS-T (3 × 5 min) before visualization with 3,3′-diaminobenzidine (DAB), and counterstained with haematoxylin. Finally, they were dehydrated in increasing concentrations of IDA, cleared in xylene (3 × 5 min) and mounted using distyrene plasticiser xylene (DPX).

### Immunofluorescent labelling and imaging

After the blocking in 10% FBS in PBS-T for 60 min at RT, sections were washed in PBS for 5 min and incubated for 1 min in TrueBlack lipofuscin autofluorescence quencher (Biotium #23,007) in 70% ethanol. The sections were washed in PBS (3 × 5 min) and incubated in primary antibodies overnight at 4 °C. They were rinsed in PBS (3 × 5 min), incubated in secondary antibodies for 1 h at RT in dark and washed in PBS (3 × 5 min). The slides were mounted using an aqueous mounting medium with DAPI (Vector Laboratories #H-1500-10). Tiled imaging was carried out on the Olympus VS120 microscope. Confocal imaging was carried out on a confocal laser-scanning microscope (LSM 700, Carl Zeiss, Germany), and image analysis on Zen Digital Imaging software (RRID: SCR_013672).

### Recombinant aSyn generation, antibody pre-adsorption and slot blot (SB) analysis

aSyn expression and purification was performed as described [17]. In brief, aSyn human WT-encoding pT7-7 plasmids were used to transform BL21(DE3) chemically competent *E. coli*, which were then grown on an agar dish supplemented with ampicillin. A single colony was transferred to Luria broth media with ampicillin at 100 µg/mL, the small culture was left to grow at 37 °C on shaker (at 180RPM) for 16 h and was then used to inoculate a large culture of 6L Luria broth media supplemented with ampicillin at 100 µg/mL. aSyn expression was induced at an optic density (OD_600_) of 0.5–0.6A, using isopropyl β-D-1-thiogalactopyranoside at a final concentration of 1 mM. The culture was grown for another 4 h on shaker, centrifuged at 4000* g* for 15 min at 4 °C, and the pellet collected. The lysis buffer of 20 mM Tris pH8.0, 0.3 µM phenylmethylsulfonyl fluoride (PMSF) protease inhibitor and cOmplete^TM^, mini, EDTA-free protease inhibitor cocktail tablet (Roche #4,693,159,001; one tablet per 10 mL lysis buffer) was used to re-suspend the pellet (10 mL p/L of culture) on ice. Cell lysis was carried out by sonication (59 s-pulse and 59 s-no pulse over 5 min at 60% amplitude), and the lysate was spun down for 30 min at 20,000 g and 4 °C. The supernatant was collected and boiled for 15 min at 100 °C, and the centrifugation repeated before the supernatant was filtered via a 0.22 µm syringe filter. The purification was performed by anion exchange chromatography and reverse-phase high performance liquid chromatography (HPLC). The protein quality control was carried out by liquid chromatography-mass spectrometry (LC–MS), ultra-performance liquid chromatography (UPLC), and SDS-PAGE separation and Coomassie staining. A semi-synthetic approach was taken for the preparation of the aSyn nY39 [18] and pY39 [19] proteins as described previously [20]. Briefly, a three-fragment one-pot chemical semi-synthesis strategy was followed. The first native chemical ligation (NCL) reaction involved the ligation of aSyn(A56C-140) with Thz-aSyn(31–55)SR pY39 or Thz-aSyn(31–55)SR nY39. This reaction generated Thz-aSyn(31–140) A56C pY39 or Thz-aSyn(31–140) A56C nY39 as the intermediate fragments, which were then ligated with aSyn(1–29)SR to yield aSyn(1–140) A30C A56C pY39 or aSyn(1–140) A30C A56C nY39. The desulphurization of the final ligation products regenerated the native alanine residues. The proteins were purified via reversed-phase high-performance liquid chromatography (RP-HPLC).

For the antibody pre-adsorption, 5-fold of recombinant aSyn protein, or just PBS as control, was added to the IHC-optimized antibody solution in PBS (see Additional file 1: Table S1 for the IHC dilutions). The mixture was incubated overnight at 4 °C on a wheel, and the probing protocol, adapted from [21], was carried out for the slot blot analysis. 200 ng of aSyn proteins diluted in PBS to 100µL were blotted on 0.22 µm nitrocellulose membranes, which were blocked at 4 °C overnight in Odyssey blocking buffer (Li-Cor). After the incubation with primary antibodies diluted in PBS for 2 h at RT, the membranes were washed × 3 for 10 min in PBS with 0.01% Tween-20 (PBS-T), incubated with the secondary antibodies diluted in PBS in the dark, and washed × 3 for 10 min in PBS-T. For the SB dilutions of the primary and secondary antibodies, see Additional file 1: Table S1. Imaging was carried out at 700 nm and 800 nm using Li-Cor Odyssey CLx, and the image processing using Image Studio 5.2.

## Results

### Mapping the astrocytic aSyn proteoform in LB disorders

**Fig. 1:**
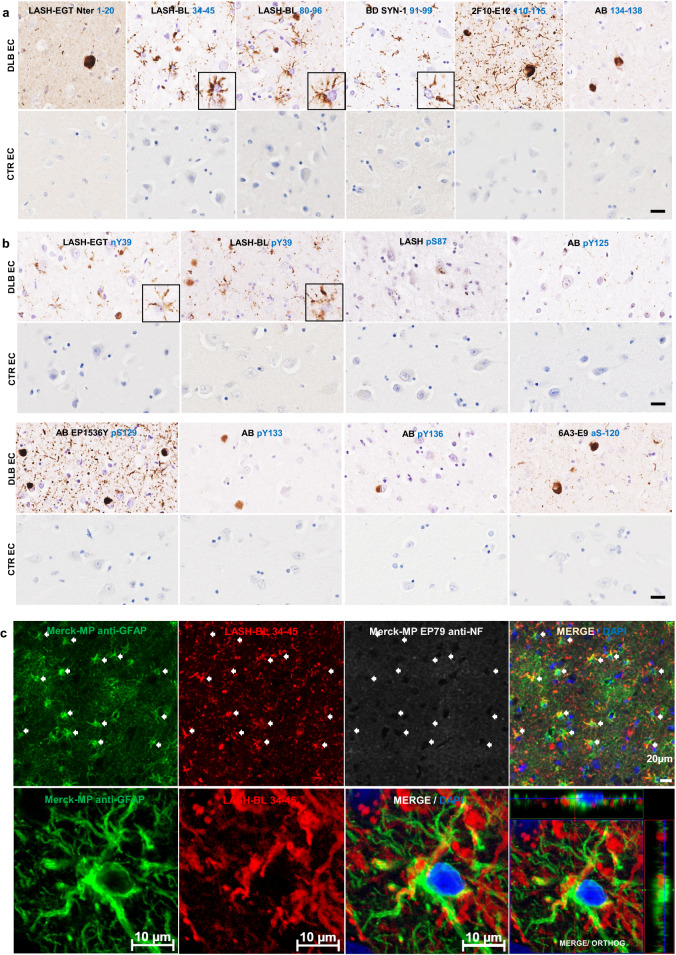
The star-like astrocytic aSyn accumulations in the DLB entorhinal cortex (EC). **a** The EC of DLB and CTR brains were immunohistochemically stained using aSyn antibodies with epitopes against the N-terminus (LASH-EGTNter 1–20 and LASH-BL 34–45), the NAC region (LASH-BL 80–96 and BD SYN-1 91–99) and the C-terminus (2F10-E12 110–115 and AB 134–138) of aSyn. The extreme N-terminal antibody LASH-EGTNter as well as the C-terminal antibodies 2F10-E12 and AB 134–138 showed neuronal pathology in the soma and neurites. The late N-terminal antibody LASH-BL 34–45 as well as the two NAC region antibodies LASH-BL 80–96 and BD SYN-1 were positive for LBs and LNs, but also distinctively detected star-shaped glial aSyn species (insets). Representative images taken from the cortical deep grey matter (layers V–VI) of DLB2 and CTR1. **b** The aSyn PTM antibodies against phosphorylation and nitration at Tyrosine 39 (Y39) were also reactive to the star-like astroglial pattern (insets). Representative images taken from the cortical deep grey matter (layers V–VI) of DLB2 and CTR2. **c** Star-like aSyn species are associated with the GFAP-positive astrocytes in the DLB brains as shown by IF using antibodies for astrocytic and neuronal markers GFAP and NF, and LASH-BL 34–45 antibody against aSyn. The star-like aSyn species (arrows) appeared in and around the GFAP-positive astrocytes, and not in the LNs. Representative images from DLB1 cingulate cortex. Images on the upper panel taken using Olympus slide scanner at 40 × magnification, and the lower panel on Zeiss LSM700 confocal microscope. Scale bar for Fig. 1a, b is 20 µm for the main images and 40 µm for the insets. aSyn = alpha-synuclein; CTR = control; DLB = dementia with Lewy bodies; EC = entorhinal cortex; GFAP = glial fibrillary acidic protein; IF = immunofluorescence; LB = Lewy body; LN = Lewy neurite; NAC = non-amyloid component; NF = neurofilament; PTM = post-translational modification

We have previously shown that the use of antibodies against different regions and post-translational modifications of aSyn enables revealing the pathological diversity across synucleinopathies [22]. Therefore, we sought to use an expanded antibody toolbox to characterize the aSyn astrocytic pathology. A complete list of the antibodies used in this study and their epitopes is shown in Additional file 1: Table S1. DLB entorhinal cortex was stained using two antibodies against the N-terminal (epitopes 1–20 and 34–45), two against the NAC region (epitopes 80–96 and 91–99) and two against the C-terminal regions (epitopes 110–115 and 134–138) of aSyn (Fig. 1a). Serial sections from the same region were also screened for aSyn post-translational modifications (PTMs), including Serine (at Ser87 and Ser129) and Tyrosine (at Tyr39, Tyr125, Tyr133 and Tyr136) phosphorylations, N-terminal nitration at Tyr39 (nY39) and C-terminal truncation at residue 120 (Fig. 1b). Whilst all the antibodies against non-modified aSyn were able to detect LBs and LNs, only the N-terminal antibody LASH-BL 34–45 and the two NAC region antibodies LASH-BL 80–96 and BD SYN-1 (epitope 91–99) were able to reveal the star-like astroglial aSyn structures (Fig. 1a). We confirmed the specificity of these three antibodies to aSyn by pre-adsorption treatment, after which the positivity to LBs and star-like structures was lost (Additional file 2: Fig. S1). Strikingly, only the two antibodies against the aSyn PTMs in the N-terminal region of the protein, i.e. pY39 and nY39, but not the antibodies targeting the C-terminal aSyn PTMs, were positive for these astroglial structures (Fig. 1b).

To validate that these structures represent aSyn in the astrocytes, we performed immunofluorescent labelling. DLB cingulate cortex was stained using LASH-BL 34–45, the best performing antibody to reveal the star-like aSyn accumulations by immunohistochemistry (Fig. 1a), and glial fibrillary acidic protein (GFAP) and neurofilament (NF) antibodies, i.e. standard markers for astrocytes and neurons, respectively. The cortical LBs were positive for NF and LASH-BL 34–45, and negative for GFAP (Additional file 2: Fig. S2a). The GFAP-positive astrocytes, on the other hand, were also positive for LASH-BL 34–45, and negative for NF by confocal imaging (Fig. 1c). The oligodendroglia and the microglia in the white matter, marked with myelin basic protein (MBP) and ionised calcium binding adaptor protein 1 (Iba1) respectively, were negative to aSyn (Additional file 2: Fig. S2b). Microglial positivity to aSyn was observed in the grey matter as rare events and did not show a star-like morphology (Additional file 2: Fig. S2b). Collectively, these observations demonstrate that the star-like structures revealed by immunohistochemistry using the late N-terminal and NAC region aSyn antibodies represent aSyn compartmentalization in astrocytes. These findings also suggest that the extreme N- and C-terminal regions of aSyn are either masked by heavy modifications, bound to other molecules or are cleaved, and thus explain why astrocytic aSyn cannot be detected with antibodies targeting these regions.

### Astrocytic aSyn is modified at Tyrosine 39

**Fig. 2:**
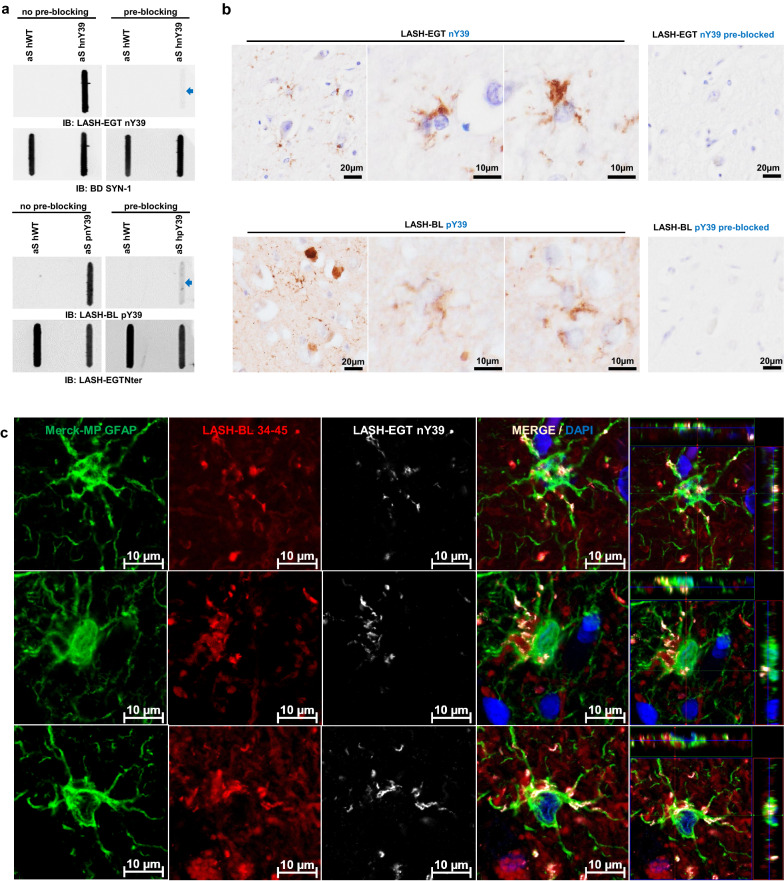
Astrocytic aSyn is modified at Tyrosine 39. **a** The specificity of the aSyn PTM antibodies against phosphorylation and nitration at Y39 were validated via antibody pre-blocking overnight followed by SB analysis. **b** The signal for astrocytic aSyn phosphorylated and nitrated at Y39 was lost with the antibody pre-blocking in the DLB1 cingulate cortex (layers V–VI). **c** The GFAP- and aSyn-positive astrocytes in the cingulate cortex of DLB1 were positive for aSyn nY39. aSyn = alpha-synuclein; DLB = dementia with Lewy bodies; GFAP = glial fibrillary acidic protein; PTM = post-translational modification; SB = slot blot

As shown in Fig. 1b, our data show for the first time that astrocytic aSyn accumulations contain a mixture of aSyn species that are phosphorylated or nitrated at Tyrosine 39 (Y39). To corroborate our findings, we first validated the specificity of the aSyn pY39 and nY39 antibodies. The antibodies were incubated with aSyn recombinant protein bearing either pY39 or nY39 before using them to detect site-specifically nitrated and phosphorylated recombinant aSyn by slot blot (SB). The positive signal for aSyn nY39 and for aSyn pY39 recombinant proteins were lost after the pre-blocking of LASH-EGT nY39 and LASH-BL pY39 antibodies, respectively (Fig. 2a). The same pre-blocking protocol was then repeated, and the pre-blocked antibody solutions applied onto DLB cingulate cortex tissues. Similarly, following the pre-blocking of the antibodies, the positivity for aSyn nY39 and pY39-positive species, including that for the astroglial structures, was abolished (Fig. 2b). Next, we immunofluorescently co-labelled the cingulate sections with GFAP and aSyn pY39 or nY39 antibodies. Consistent with our observations by brightfield microscopy (Fig. 1b), GFAP-positive astrocytes showed star-like aSyn inclusions positive for nY39 (Fig. 2c).

### Astrocytic aSyn accumulations occur across LB disorders and may be truncated in the N- and C-termini

**Fig. 3:**
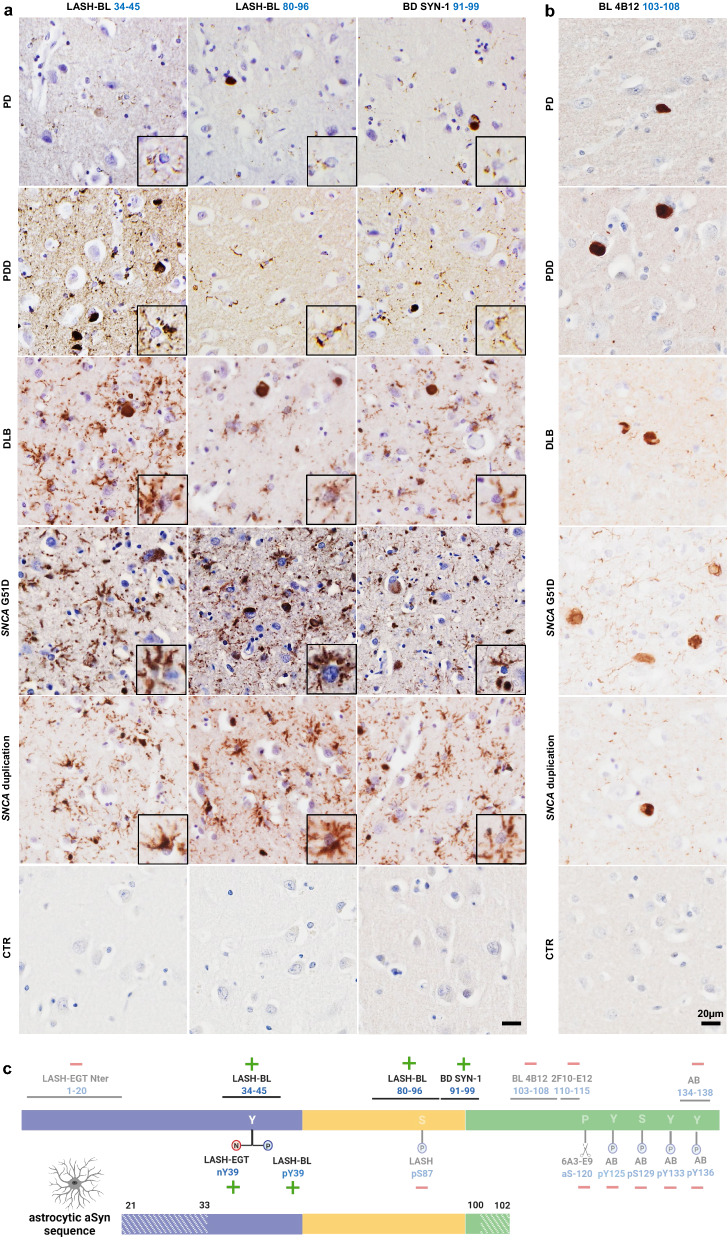
Astrocytic aSyn accumulations occur across LB disorders and may be truncated in the N- and C-termini. **a** CTR, PD, PDD, DLB, *SNCA* G51D mutation and *SNCA* duplication cingulate cortices were immunohistochemically stained using three aSyn antibodies, LASH-BL 34–45, LASH-BL 80–96 and BD SYN-1, and astrocytic accumulations (insets) were revealed across these LB disorders. **b** To further map the C-terminal truncation region of the astrocytic aSyn, the same cingulate cortex sections were stained using the C-terminal BL 4B12 antibody with an epitope 103–108 of aSyn. Neuronal inclusions were revealed, but the astrocytic aSyn accumulation was not detected, suggesting that the aSyn species associated with the astrocytes are truncated at residues 21–33 in the N-terminus, and at residues 100–102 in the C-terminus. Representative images in Fig. 3a, b taken from the cortical deep grey matter (layers V–VI) of CTR1, PD2, PDD2, DLB1, *SNCA* G51D3 and *SNCA* duplication. **c** A diagram to show the antibodies that are positive and negative for astrocytic aSyn, and their epitopes. The areas in stripes denote the potential truncation regions in the N- and C-termini. Schematic created with BioRender.com (agreement no: *DJ23GJF70T*). Scale bar for Fig. 3a is 20 µm for the main images and 40 µm for the insets. aSyn = alpha-synuclein; CTR = control; DLB = dementia with Lewy bodies; LB = Lewy body; PD = Parkinson’s disease; PDD = Parkinson’s disease with dementia

Having established that the star-like aSyn structures are astrocytic in DLB brains, we sought to explore if the astrocytic aSyn exhibits similar staining properties across other LB disorders. We screened, using the same set of antibodies against non-modified aSyn, the cingulate cortices of PD, PDD and DLB cases. We included *SNCA* G51D mutation and duplication cases in this screening to investigate if astrocytic aSyn pathology occurs in familial cases as well as in sporadic synucleinopathies. The astrocytic aSyn accumulations were observed widely across these LB diseases (Fig. 3a; Additional file 2: Fig. S3; Table 2). These sections were double-labelled with aSyn LASH-BL 34–45 antibody and GFAP, which revealed that GFAP-positive astrocytes are positive to the aSyn accumulations in these cases (Additional file 2: Fig. S4). The pons, putamen, cerebellum, frontal cortex and occipital cortex of MSA cases were stained using these aSyn antibodies, but we failed to detect any astroglial pathology in the MSA brains.

To determine if the astrocytic aSyn species are also N-terminally and/or C-terminally truncated, and to more precisely map their sequence, we stained serial cingulate sections using BL 4B12 antibody, which targets residues 103–108. Interestingly, the astroglial structures were not detected using this antibody in any of the LB disorder cases in the cingulate cortex (Fig. 3b). Our results suggest that astrocytic aSyn may be truncated in the N-terminus between residues 21–33, and in the C-terminus between residues 100–102 (Fig. 3c).

### Astrocytic aSyn accumulations are not immunoreactive for the canonical aSyn aggregation markers

**Fig. 4:**
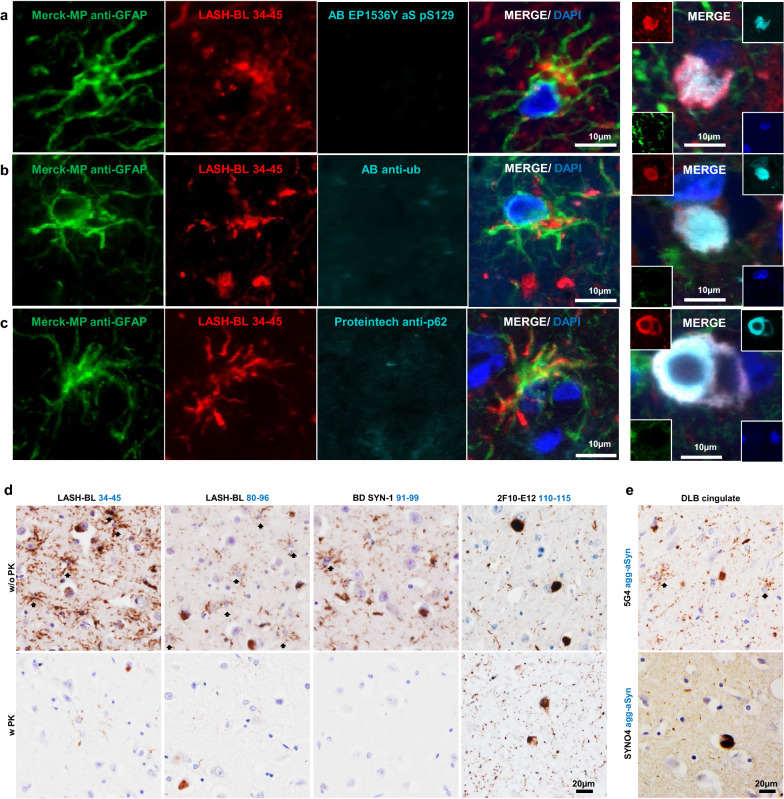
Astrocytic aSyn accumulations are free of the canonical aSyn aggregation markers. **a-c** The GFAP-positive astrocytic accumulations in DLB1 cingulate cortex were negative to aSyn pS129 (AB EP1536Y), ubiquitin and p62. Images of cortical LBs are included as positive controls for aSyn pS129, ubiquitin and p62 reactivity. Images for Fig. 4a, c taken using Olympus slide scanner at 40 × magnification. **d** The astrocytic aSyn signal (arrows) was largely abolished after PK treatment in DLB cingulate cortex. The 2F10-E12 staining was included as a positive control to show the PK resistance of LBs and LNs. Images taken from the cortical deep grey matter (layers V–VI) DLB1 cingulate cortex. **e** The star-shaped astrocytic aSyn accumulations were revealed by the 5G4 antibody, but not by the SYNO4 antibody in the DLB cingulate cortex. Representative images from the cortical deep grey matter (layers V–VI) of DLB1 and DLB3 cingulate cortex. agg-aSyn = aggregated alpha-synuclein; aSyn = alpha-synuclein; DLB = dementia with Lewy bodies; GFAP = glial fibrillary acidic protein; LB = Lewy body; LN = Lewy neurite; PK = proteinase K

To investigate the nature and aggregation state of aSyn in these accumulations, the astrocytic aSyn species were screened for the classical markers of aSyn aggregation and inclusion formation. DLB cingulate cortex was triple labelled with LASH-BL 34–45 and GFAP, and either with antibodies against p62, ubiquitin or aSyn pS129. In line with our brightfield microscopy results (Fig. 1b), the cortical LBs and LNs showed strong positivity to aSyn pS129, whereas the astrocytes positive for LASH-BL 34–45 remained negative for aSyn pS129 (Fig. 4a). Similarly, whereas the cortical LBs were positive for ubiquitin and p62, the aSyn-positive astrocytes were negative for these aggregate markers (Fig. 4b, c). Considering that several of the key lysine residues, found to be ubiquitinated in LBs [23], reside in the N-terminal domain of the protein (i.e. K12, K21 and K23), and that p62 is a monoubiquitin- and polyubiquitin-binding protein [24–26], the absence of ubiquitin and p62 positivity in the astrocytic aSyn is in line with our observations that the aSyn species in the astroglia may be N-terminally truncated.

One of the characteristics of aggregated aSyn in LB diseases is their resistance to proteinase K (PK) digestion [27, 28]. To further characterize the aggregation state of aSyn in astrocytes, we treated the DLB cingulate cortex tissues with PK, and observed that the large majority of the astrocytic aSyn signal disappeared after PK treatment (Fig. 4d). Next, we profiled the astrocytic aSyn using two antibodies, 5G4 and SYNO4, that show preferential binding to aggregated aSyn [11, 12, 21, 29]. Interestingly, the star-shaped astrocytic aSyn accumulations were revealed by the 5G4 antibody, but not by the SYNO4 antibody (Fig. 4e). Altogether, these observations suggest that aSyn species that accumulate in the astrocytes may not possess the amyloid-like properties of aSyn fibrils found in LBs and LNs, but could still represent a mixture of soluble and non-amyloidogenic aggregates, i.e. oligomers. Unfortunately, the lack of oligomer-specific antibodies and our inability to isolate and interrogate astrocytic aSyn make it difficult to more precisely determine the exact aggregation state of aSyn in the astrocytes.

### Astrocytic aSyn accumulations occur in several limbic regions of LB disorders

**Fig. 5:**
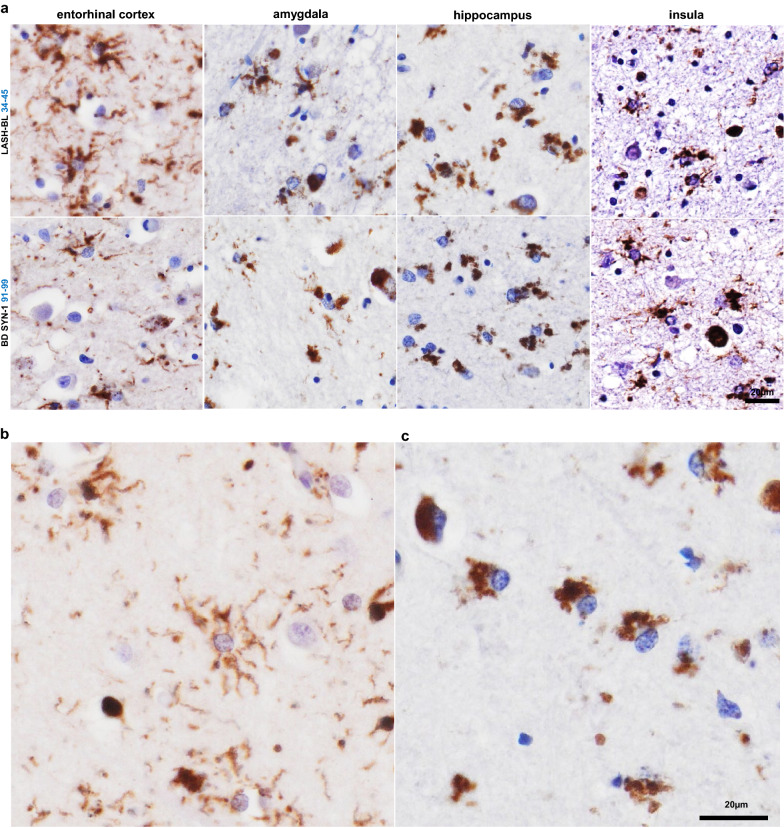
Astrocytic aSyn accumulations occur in several limbic regions of LB disease brains. **a** The astrocytic aSyn accumulations were encountered in the EC (*SNCA* G51D3), amygdala (DLB1), hippocampal CA4 (*SNCA* duplication) and insula (PDD5) regions of LB disorders. **b** The astrocytic accumulations showed morphological diversity, with the majority showing a star shape and labelling the ramified processes (cingulate cortex layers V–VI). **c** Some of the astrocytic aSyn appeared as cytoplasmic accumulations (hippocampal CA4). Figure 5b, c images from a *SNCA* duplication brain stained with the LASH-BL 34–45 antibody. aSyn = alpha-synuclein; CA4 = cornu ammonis 4; DLB = dementia with Lewy bodies; EC = entorhinal cortex; LB = Lewy body; PD = Parkinson’s disease; PDD = Parkinson’s disease with dementia

After having observed the frequent occurrence of astrocytic aSyn in the cingulate cortices of LB disorders (Fig. 3a), we expanded our screening of astrocytic aSyn species in other limbic brain regions. We observed that the astrocytic aSyn accumulations were also prominently present in the entorhinal cortex, the insula, the amygdala and the hippocampus (Fig. 5a; Additional file 2: Fig. S5) of LB disorders. Interestingly, we identified different morphologies of astrocytic aSyn accumulations. The majority of the astrocytic aSyn accumulations appeared in soma-sparing star-like forms typically labelling the ramified processes of astrocytes (Fig. 5b). In the hippocampal subregions, the astrocytic aSyn accumulations were predominantly in the soma and did not exhibit a star-shaped morphology (Fig. 5c). Altogether, our findings demonstrate that astrocytic aSyn is a prominent pathological feature of LB diseases and presents itself in several brain regions.

## Discussion

**Fig. 6:**
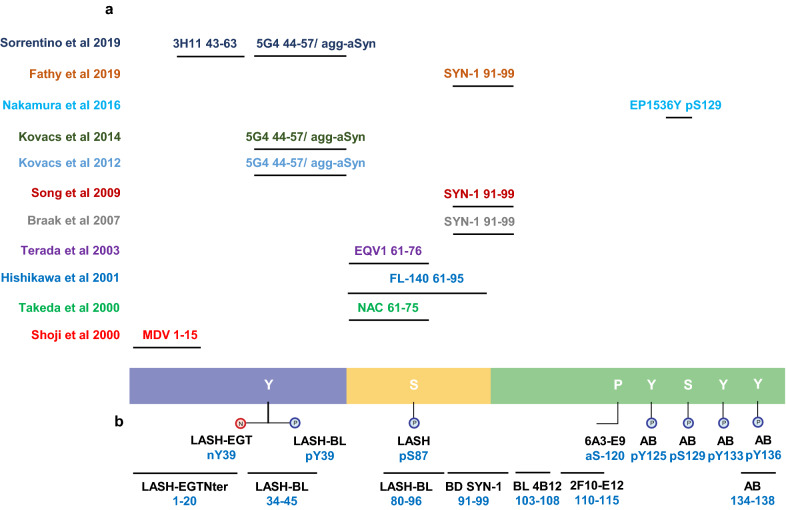
**a** A diagrammatic representation of the antibodies used in the previous publications reporting aSyn positivity in the astrocytes (only the studies using antibodies with a specified epitope are included). **b** The antibodies used in this study. aSyn = alpha-synuclein; agg-aSyn = aggregated alpha-synuclein

Astrocytic aSyn pathology is a relatively less explored aspect of neuropathology in the synucleinopathies. In this study, we systematically characterized the sequence and aggregation state of astrocytic aSyn accumulations using antibodies against non-modified and post-translationally modified forms of aSyn across the Lewy body diseases. Our results demonstrate that the astrocytic aSyn accumulations are widely present in several brain regions of PD, PDD and DLB cases, are negative for ubiquitin, p62 and aSyn pS129, but show positivity to other aSyn PTMs, including nitration and phosphorylation at Y39. Furthermore, only a subset of antibodies against non-modified aSyn are able to reveal astrocytic aSyn in brain tissues. These are antibodies that targeted the NAC (80–96 and 91–99) and the late N-terminal (34–45) regions of the protein. This is in line with the previous studies that reported astrocytic positivity only, or primarily with NAC region aSyn antibodies [4, 6, 7, 9–12, 14, 15] (Fig. 6a). However, our studies allow for the more precise mapping, to the extent possible using antibodies, of the potential cleavage sites and demonstrate that the majority of astrocytic aSyn is likely both N- and C-terminally truncated (Fig. 6b). In addition, we demonstrate for the first time that astrocytic aSyn exists as a mixture of non-amyloid species that are phosphorylated or nitrated at Y39.

The fact that we were not able to detect astrocytic aSyn species with three antibodies targeting the C-terminal region of the protein spanning residues 103 to 138 strongly suggests that the astrocytic aSyn may be C-terminally truncated somewhere between residues 100–102. Similarly, the astrocytic aSyn species were detected by an antibody targeting residues 34–45 (LASH-BL 34–45), but not an antibody targeting the first N-terminal 20 amino acids (LASH-EGTNter 1–20). These results suggest that the N-terminal truncation of astrocytic aSyn is likely to occur between residues 21–33. Although an initial study [5] reported the detection of astrocytic aSyn using an antibody with an N-terminal epitope (MDV, 1–15), subsequent studies showed that N-terminal antibodies covering aSyn residues 1–21 [6, 7, 15] did not detect astrocytic aSyn. Altogether, these data demonstrate that the great majority of aSyn in astrocytes may be subjected to both N- and C-terminal cleavage at approximately residues 21–33 and 100–102, respectively. These results are supported by our findings that astrocytic aSyn inclusions were immunoreactive for only aSyn PTMs in the mid-N-terminal region. However, we cannot rule out the possibility that the extreme N- and C-terminal sequences are masked by aSyn interactions with other proteins, especially since both termini serve as hubs for regulating aSyn membranes/lipids and protein interactions. If this is the case, it would suggest that aSyn conformations and interactome in astrocytes are distinct from those in neurons, where aSyn is detectable using both N- and C-terminal-targeting antibodies. Whether truncated aSyn species are cleaved in the neurons, in the astrocytes or in the extracellular space is an important gap of knowledge that should be addressed and could shed new light into the role of PTMs in regulating the function/dysfunction of aSyn and mechanisms of aSyn trafficking between neurons and glia in the disease brains.

Very little is known about the aggregation state of the astrocytic aSyn species. Kovacs and colleagues [11, 12] were the first to show positivity for astrocytes with the 5G4 antibody, reported to be specific for oligomeric and fibrillar forms of aSyn [12, 21]. Similar astrocytic positivity using 5G4 was also detected by Sorrentino and colleagues [15], but there has not been any studies to validate these findings using multiple aSyn aggregate-specific antibodies or to define the aggregation state of aSyn in astrocytes. Similarly, only two studies have assessed the ultrastructure of astrocytic aSyn by EM [11, 13]. Nakamura et al. described the aSyn pS129-positive subpial astrocytic processes in the MSA brains as non-filamentous [13], and Kovacs and colleagues reported that the astrocytic accumulations of LB diseases are beta-sheet-rich oligomers [11].

In this study, we investigated the aggregation state of aSyn in astrocytes using multiple approaches, including aSyn conformational/ aggregate-specific antibodies (5G4 and SYNO4) and limited proteolysis (PK resistance). In line with Kovacs and colleagues [11], the astrocytic inclusions of LB diseases were revealed by 5G4, but were not detected with SYNO4. We also report that the majority of the astrocytic aSyn did not show resistance to PK digestion. These findings, combined with our observation that astrocytic aSyn accumulations are not positive for the canonical markers of LBs, including ubiquitin, p62, and the most common pathology-associated aSyn PTM, pS129 [23, 30], suggest that aSyn accumulations in astrocytes possess a distinct PTM and sequence signature, and may be composed primarily of oligomers or other non-fibrillar forms of the protein.

We speculate that the lack of aSyn phosphorylation at S129 could be because the astrocytic aSyn species are truncated in the C-terminus, and no longer carry the binding site for the aSyn pS129 antibodies. Likewise, these aSyn accumulations may be cleaved in the N-terminus, the domain where aSyn is found to be ubiquitinated in the disease brains [23]. Given that aSyn pS129 has been reported to be important for priming aSyn ubiquitination [31], the absence of pS129 could explain the absence of ubiquitination at other lysine residues in the protein. One final possibility is that both of these aSyn PTMs are linked to the formation of aSyn pathology [23, 30, 31], and their absence suggests that the astrocytic aSyn species exist in non-aggregated forms.

It is important to note that while Nakamura et al. observed non-filamentous aSyn pS129 accumulation in the subpial and periventricular astrocytes in the spinal cord and subependymal area of the lateral ventricle in 6/15 (40%) MSA patients [13], we failed to detect any astrocytic aSyn positivity in the MSA cases studied. This discrepancy in findings may be due to the differences between the regions looked at as well as the number and disease duration of the cases included in each study. We aim to further assess the presence of astrocytic aSyn in a larger cohort of MSA cases covering a greater number of brain regions.

The fact that astrocytic aSyn appears to be cleaved and non-fibrillar at the same time is surprising given that removal of the solubilizing N- and C-terminal domains is expected to increase the hydrophobicity and aggregation propensity of the protein [32–37]. Therefore, more extensive investigations of astrocytic aSyn conformations and aggregation state are needed. These studies could shed novel insights into the function(s) of aSyn in astrocytes and the role of astrocytic pathology in the pathogenesis of LB diseases. Furthermore, understanding what may be keeping these truncated aSyn species from forming fibrils in astrocytes could shed light on novel mechanisms for regulating aSyn aggregation.

These observations raise important questions about the origins and mechanisms involved in the astrocytic uptake, processing, degradation and/or release of aSyn. Cell culture studies have shown that astrocytes take up [38–44], degrade [40, 42–44], and/or release [39, 43, 44] aSyn. Yet, a consensus has not been reached on whether or not the astrocytic uptake and processing of aSyn may have cytoprotective [40, 42, 43] or cytotoxic [38, 39, 41, 42, 44] consequences. Kovacs and colleagues have shown that the astrocytic aSyn is localized in the endo-lysosomal compartments in the disease brains [11]. Similarly, cell model-based studies has shown that glial-glial and glial-neuronal oligomeric aSyn transfer can occur in lysosomal vesicles via direct transfer or tunnelling nanotubes [39, 43, 44]. The possibility that the great majority, if not all, of astrocytic aSyn across different LBDs is truncated suggests differential processing of aSyn in the astrocytes that may reflect its astrocytic functions, or a cellular response to aSyn species originating neurons or other glial cells. Altogether, a more precise understanding of the astrocytic involvement in the cell-to-cell propagation of misfolded aSyn is needed and could provide novel insights into the mechanisms and pathways underpinning pathology spreading in LB diseases.

Astrocytes are the most populous type of glial cells in the brain, with crucial functions in neuronal survival, synaptic maintenance, glucose metabolism, water homeostasis and in immune response [45]. Insults may activate astrocytes [46], which can in turn signal the microglia [47–49] and act as key determinants of microglial activation and neuroinflammation in disease progression [50]. Furthermore, aSyn aggregates have been reported to activate both astrocytes [41, 51–54] and microglia [53, 55] into giving an inflammatory response. Nitration of aSyn in particular has been reported to induce microglial activation [56–59], which may be responsible for some of its neurotoxic properties [57, 58]. We found astrocytic aSyn to be nitrated at Y39 and speculate that this specific aSyn PTM may play a key role in the astrocytic signalling of microglia and neuroinflammation in LB diseases. Further studies to investigate the mechanisms of astrocytic activation of microglia, and the involvement of aSyn nY39 taken up and/or released by astrocytes within this context may further explain the interaction of neuroinflammation and neurodegeneration in LB disorders.

To our knowledge, this is the first study that examined the post-translational modifications profile (serine and tyrosine phosphorylations, tyrosine nitration and N- and C-terminal truncations) of astrocytic aSyn inclusions in LB disorders. Although this hypothesis cannot be validated by biochemical profiling due to technical challenges associated with the isolation of astrocytic accumulations from the rest of the aSyn pathology, the failure of four different N- and C-terminal antibodies to detect astrocytic aSyn species supports our conclusions on the sequence properties of aSyn in astrocytic pathology. Furthermore, this is the first study reporting on aSyn brain pathological species that appears to be composed primarily of truncated aSyn. Previous studies have also shown high abundance of N- and C-terminally truncated aSyn species in the human brain [23, 60–63] and appendix [64]; however, in many of these studies the full-length protein remains highly abundant as the dominant species, as evidenced by the fact that antibodies against phosphorylated aSyn at S129 remain the primary tools used to monitor and quantify aSyn pathology in human brains and in animal models of synucleinopathies. The possible co-occurrence of N- and C-terminal truncation in the astrocytic aSyn without fibrillization is intriguing, as the NAC region alone is known to be highly prone to aggregation by itself [35]. Understanding which cell-specific mechanisms may prevent aSyn from forming fibrils in the astrocytes could have important implications for understanding the cellular determinants of aSyn pathology formation and developing more effective strategies to prevent aSyn aggregation and pathology formation.

Our findings raise several important questions and call for further studies to clarify (1) if and where the astrocytic aSyn is N- and C-terminally truncated using more precise, proteomics-based approaches, perhaps in combination with laser capture microscopy technique; (2) if these truncated species of aSyn become cleaved after internalization by the astrocytes, or are internalized after being cleaved in neurons; (3) why these astrocytic aSyn inclusions appear in abundance in LBDs but are spared in MSA; (4) the precise nature of the aggregation state of aSyn in these astrocytes; and (5) the occurrence of astrocytic pathology in relation to LB disease staging, clinical progression and clinical phenotypes. The expanded toolset that we present here should facilitate these studies and advance our understanding of the function of astrocytic aSyn in health and disease.

## Supplementary Information

**Additional file 1**: **Table S1**. The primary and secondary antibodies included in this study. AC = autoclave; agg-aSyn = aggregated alpha-synuclein; aSyn = alpha-synuclein; FA = formic acid; GFAP = glial fibrillary acidic protein; Iba1 = ionized calcium binding adaptor protein 1; IF = immunofluorescence; IHC = immunohistochemistry; MBP = myelin basic protein; mc = monoclonal; mus = mouse; na = not applicable; NF = neurofilament; pc = polyclonal; rab = rabbit; SB = slot blot.

**Additional file 2**: **Fig. S1**. Specificity validation of the aSyn antibodies LASH-BL 34-45, LASH-BL 80-96 and BD SYN-1 by pre-adsorption followed by IHC on DLB1 cingulate cortex (layers V–VI). aSyn = alpha-synuclein; DLB = dementia with Lewy bodies; IHC = immunohistochemistry. **Fig. S2**. (a) A representative image of a cortical LB positive for NF and LASH-BL 34-45, and negative for GFAP. Image from DLB1 cingulate cortex taken using Zeiss LSM700 confocal microscope. (b) The oligodendrocytes and microglial cells, marked by anti-MBP and anti-Iba1 antibodies, respectively, were negative for aSyn in the white matter (upper two panels). Punctate aSyn positivity was detected in the microglial cells in the grey matter (lower panel) as rare events. Images taken from DLB1 cingulate cortex using Olympus slide scanner at 40x magnification. aSyn = alpha-synuclein; DLB = dementia with Lewy body; GFAP = glial fibrillary acidic protein; Iba1 = ionised calcium binding adaptor protein 1; MBP = myelin basic protein; LB = Lewy body; NF = neurofilament. **Fig. S3**. The cingulate cortex of sporadic PD, PDD, DLB, SNCA G51D mutation and SNCA duplication cases immunostained using antibodies against the N-terminal (LASH-EGTNter) and C-terminal (2F10-E12 and AB 134-138) of aSyn. Astrocytic aSyn was not detected using these antibodies. Representative images taken from cingulate cortical layers V–VI of PD7, PDD5, DLB1, SNCA G51D3 and SNCA duplication cases. aSyn = alpha-synuclein; DLB = dementia with Lewy bodies; PD = Parkinson’s disease; PDD = Parkinson’s disease with dementia. **Fig. S4**. Representative IF images of GFAP-positive astrocytes from DLB1, SNCA G51D3 and SNCA duplication cingulate cortices, showing positivity for aSyn detected using LASH-BL 34-45 antibody. Images taken using Olympus slide scanner at 40x magnification. aSyn = alpha-synuclein; DLB = dementia with Lewy body; GFAP = glial fibrillary acidic protein; IF = immunofluorescence; NF = neurofilament. **Fig. S5**. Representative images from the SNCA duplication EC, amygdala and insula immunostained using antibodies against the N-terminal, NAC and C-terminal regions of aSyn. The astrocytic aSyn detected only by LASH-BL34-45, LASH-BL80-96 and BD SYN-1 (91-99) antibodies. Similar staining patterns were observed in the same regions from PD, PDD, DLB and SNCA G51D cases stained with the same antibody set. aSyn = alpha-synuclein; DLB = dementia with Lewy bodies

## References

[1] Baba M, Nakajo S, Tu PH, Tomita T, Nakaya K, Lee VM, Trojanowski JQ, Iwatsubo T (1998). Aggregation of alpha-synuclein in Lewy bodies of sporadic Parkinson’s disease and dementia with Lewy bodies. Am J Pathol.

[2] Spillantini MG, Crowther RA, Jakes R, Hasegawa M, Goedert M (1998). Alpha-synuclein in filamentous inclusions of Lewy bodies from Parkinson’s disease and dementia with Lewy bodies. Proc Natl Acad Sci.

[3] Spillantini MG, Crowther RA, Jakes R, Cairns NJ, Lantos PL, Goedert M (1998) Filamentous a-synuclein inclusions link multiple system atrophy with Parkinson’s disease and dementia with Lewy bodies. Neurosci Lett 4 10.1016/s0304-3940(98)00504-7 9726379

[4] Hishikawa N, Hashizume Y, Yoshida M, Sobue G (2001). Widespread occurrence of argyrophilic glial inclusions in Parkinson’s disease. Neuropathol Appl Neurobiol.

[5] Shoji M, Harigaya Y, Sasaki A, Ueda K, Ishiguro K, Matsubara E, Watanabe M, Ikeda M, Kanai M, Tomidokoro Y, Shizuka M, Amari M, Kosaka K, Nakazato Y, Okamoto K, Hirai S (2000). Accumulation of NACP/alpha -synuclein in Lewy body disease and multiple system atrophy. J Neurol Neurosurg Psychiatry.

[6] Takeda A, Hashimoto M, Mallory M, Sundsumo M, Hansen L, Masliah E (2000). C-terminal α-synuclein immunoreactivity in structures other than Lewy bodies in neurodegenerative disorders. Acta Neuropathol.

[7] Terada S, Ishizu H, Yokota O, Tsuchiya K, Nakashima H, Ishihara T, Fujita D, Uéda K, Ikeda K, Kuroda S (2003). Glial involvement in diffuse Lewy body disease. Acta Neuropathol.

[8] Wakabayashi K, Hayashi S, Yoshimoto M, Kudo H, Takahashi H (2000). NACP/α-synuclein-positive filamentous inclusions in astrocytes and oligodendrocytes of Parkinson’s disease brains. Acta Neuropathol.

[9] Braak H, Sastre M, Del Tredici K (2007). Development of α-synuclein immunoreactive astrocytes in the forebrain parallels stages of intraneuronal pathology in sporadic Parkinson’s disease. Acta Neuropathol.

[10] Fathy YY, Jonker AJ, Oudejans E, Jong FJJ, Dam A-MW, Rozemuller AJM, Berg WDJ (2019). Differential insular cortex subregional vulnerability to α-synuclein pathology in Parkinson’s disease and dementia with Lewy bodies. Neuropathol Appl Neurobiol.

[11] Kovacs GG, Breydo L, Green R, Kis V, Puska G, Lőrincz P, Perju-Dumbrava L, Giera R, Pirker W, Lutz M, Lachmann I, Budka H, Uversky VN, Molnár K, László L (2014). Intracellular processing of disease-associated α-synuclein in the human brain suggests prion-like cell-to-cell spread. Neurobiol Dis.

[12] Kovacs GG, Wagner U, Dumont B, Pikkarainen M, Osman AA, Streichenberger N, Leisser I, Verchère J, Baron T, Alafuzoff I, Budka H, Perret-Liaudet A, Lachmann I (2012). An antibody with high reactivity for disease-associated α-synuclein reveals extensive brain pathology. Acta Neuropathol.

[13] Nakamura K, Mori F, Kon T, Tanji K, Miki Y, Tomiyama M, Kurotaki H, Toyoshima Y, Kakita A, Takahashi H, Yamada M, Wakabayashi K (2016). Accumulation of phosphorylated α-synuclein in subpial and periventricular astrocytes in multiple system atrophy of long duration: phosphorylated α-synuclein in MSA astrocytes. Neuropathology.

[14] Song YJC, Halliday GM, Holton JL, Lashley T, O’Sullivan SS, McCann H, Lees AJ, Ozawa T, Williams DR, Lockhart PJ, Revesz TR (2009). Degeneration in different Parkinsonian syndromes relates to astrocyte type and astrocyte protein expression. J Neuropathol Exp Neurol.

[15] Sorrentino ZA, Goodwin MS, Riffe CJ, Dhillon J-KS, Xia Y, Gorion K-M, Vijayaraghavan N, McFarland KN, Golbe LI, Yachnis AT, Giasson BI (2019). Unique α-synuclein pathology within the amygdala in Lewy body dementia: implications for disease initiation and progression. Acta Neuropathol Commun.

[16] Terada S, Ishizu H, Haraguchi T, Takehisa Y, Tanabe Y, Kawai K, Kuroda S (2000). Tau-negative astrocytic star-like inclusions and coiled bodies in dementia with Lewy bodies. Acta Neuropathol.

[17] Fauvet B, Mbefo MK, Fares M-B, Desobry C, Michael S, Ardah MT, Tsika E, Coune P, Prudent M, Lion N, Eliezer D, Moore DJ, Schneider B, Aebischer P, El-Agnaf OM, Masliah E, Lashuel HA (2012). α-Synuclein in central nervous system and from erythrocytes, mammalian cells, and *Escherichia coli* exists predominantly as disordered monomer. J Biol Chem.

[18] Burai R, Ait-Bouziad N, Chiki A, Lashuel HA (2015). Elucidating the role of site-specific nitration of α-synuclein in the pathogenesis of Parkinson’s disease via protein semisynthesis and mutagenesis. J Am Chem Soc.

[19] Dikiy I, Fauvet B, Jovičić A, Mahul-Mellier A-L, Desobry C, El-Turk F, Gitler AD, Lashuel HA, Eliezer D (2016). Semisynthetic and in vitro phosphorylation of alpha-synuclein at Y39 promotes functional partly helical membrane-bound states resembling those induced by PD mutations. ACS Chem Biol.

[20] Fauvet B, Butterfield SM, Fuks J, Brik A, Lashuel HA (2013). One-pot total chemical synthesis of human α-synuclein. Chem Commun.

[21] Kumar ST, Jagannath S, Francois C, Vanderstichele H, Stoops E, Lashuel HA (2020). How specific are the conformation-specific α-synuclein antibodies? Characterization and validation of 16 α-synuclein conformation-specific antibodies using well-characterized preparations of α-synuclein monomers, fibrils and oligomers with distinct structures and morphology. Neurobiol Dis.

[22] Altay MF, Kumar ST, Burtscher J, Jagannath S, Strand C, Miki Y, Parkkinen L, Holton JL, Lashuel HA (2022) Development and validation of an expanded antibody toolset that captures alpha-synuclein pathological diversity in Lewy body diseases. bioRxiv 2022.05.26.493598 10.1038/s41531-023-00604-y PMC10703845 38062007

[23] Anderson JP, Walker DE, Goldstein JM, de Laat R, Banducci K, Caccavello RJ, Barbour R, Huang J, Kling K, Lee M, Diep L, Keim PS, Shen X, Chataway T, Schlossmacher MG, Seubert P, Schenk D, Sinha S, Gai WP, Chilcote TJ (2006). Phosphorylation of Ser-129 is the dominant pathological modification of α-synuclein in familial and sporadic Lewy body disease. J Biol Chem.

[24] Cavey JR, Ralston SH, Hocking LJ, Sheppard PW, Ciani B, Searle MS, Layfield R (2004). Loss of ubiquitin-binding associated with Paget’s disease of bone p62 (SQSTM1) mutations. J Bone Miner Res.

[25] Lee Y, Weihl CC (2017). Regulation of SQSTM1/p62 via UBA domain ubiquitination and its role in disease. Autophagy.

[26] Raasi S, Varadan R, Fushman D, Pickart CM (2005). Diverse polyubiquitin interaction properties of ubiquitin-associated domains. Nat Struct Mol Biol.

[27] Neumann M, Müller V, Kretzschmar HA, Haass C, Kahle PJ (2004). Regional distribution of proteinase K-resistant α-synuclein correlates with Lewy body disease stage. J Neuropathol Exp Neurol.

[28] Tanji K, Mori F, Mimura J, Itoh K, Kakita A, Takahashi H, Wakabayashi K (2010). Proteinase K-resistant α-synuclein is deposited in presynapses in human Lewy body disease and A53T α-synuclein transgenic mice. Acta Neuropathol.

[29] Vaikath NN, Majbour NK, Paleologou KE, Ardah MT, van Dam E, van de Berg WDJ, Forrest SL, Parkkinen L, Gai W-P, Hattori N, Takanashi M, Lee S-J, Mann DMA, Imai Y, Halliday GM, Li J-Y, El-Agnaf OMA (2015). Generation and characterization of novel conformation-specific monoclonal antibodies for α-synuclein pathology. Neurobiol Dis.

[30] Fujiwara H, Hasegawa M, Dohmae N, Kawashima A, Masliah E, Goldberg MS, Shen J, Takio K, Iwatsubo T (2002). α-Synuclein is phosphorylated in synucleinopathy lesions. Nat Cell Biol.

[31] Hasegawa M, Fujiwara H, Nonaka T, Wakabayashi K, Takahashi H, Lee VM-Y, Trojanowski JQ, Mann D, Iwatsubo T (2002). Phosphorylated α-synuclein is ubiquitinated in α-synucleinopathy lesions. J Biol Chem.

[32] Bodles AM, Guthrie DJS, Harriott P, Campbell P, Irvine GB (2000). Toxicity of non-Aβ component of Alzheimer’s disease amyloid, and N-terminal fragments thereof, correlates to formation of β-sheet structure and fibrils: toxicity of non-Aβ component and fragments thereof. Eur J Biochem.

[33] Crowther RA, Jakes R, Spillantini MG, Goedert M (1998). Synthetic filaments assembled from C-terminally truncated α-synuclein. FEBS Lett.

[34] Eliezer D, Kutluay E, Bussell R, Browne G (2001). Conformational properties of α-synuclein in its free and lipid-associated states 1 1edited by P E Wright. J Mol Biol.

[35] Giasson BI, Murray IVJ, Trojanowski JQ, Lee VM-Y (2001). A hydrophobic stretch of 12 amino acid residues in the middle of α-synuclein is essential for filament assembly. J Biol Chem.

[36] Han H, Weinreb PH, Lansbury PT (1995). The core Alzheimer’s peptide NAC forms amyloid fibrils which seed and are seeded by β-amyloid: is NAC a common trigger or target in neurodegenerative disease?. Chem Biol.

[37] Volpicelli-Daley LA, Luk KC, Patel TP, Tanik SA, Riddle DM, Stieber A, Meaney DF, Trojanowski JQ, Lee VM-Y (2011). Exogenous α-synuclein fibrils induce Lewy body pathology leading to synaptic dysfunction and neuron death. Neuron.

[38] Braidy N, Gai W-P, Xu YH, Sachdev P, Guillemin GJ, Jiang X-M, Ballard JWO, Horan MP, Fang ZM, Chong BH, Chan DY (2013). Uptake and mitochondrial dysfunction of alpha-synuclein in human astrocytes, cortical neurons and fibroblasts. Transl Neurodegener.

[39] Cavaliere F, Cerf L, Dehay B, Ramos-Gonzalez P, De Giorgi F, Bourdenx M, Bessede A, Obeso JA, Matute C, Ichas F, Bezard E (2017). In vitro α-synuclein neurotoxicity and spreading among neurons and astrocytes using Lewy body extracts from Parkinson disease brains. Neurobiol Dis.

[40] Hua J, Yin N, Xu S, Chen Q, Tao T, Zhang J, Ding J, Fan Y, Hu G (2019). Enhancing the astrocytic clearance of extracellular α-synuclein aggregates by Ginkgolides attenuates neural cell injury. Cell Mol Neurobiol.

[41] Lee H-J, Suk J-E, Patrick C, Bae E-J, Cho J-H, Rho S, Hwang D, Masliah E, Lee S-J (2010). Direct transfer of α-synuclein from neuron to astroglia causes inflammatory responses in synucleinopathies*. J Biol Chem.

[42] Lindstrom V, Gustafsson G, Sanders LH, Howlett EH, Sigvardson J, Kasrayan A, Ingelsson M, Bergström J, Erlandsson A (2017). Extensive uptake of α-synuclein oligomers in astrocytes results in sustained intracellular deposits and mitochondrial damage. Mol Cell Neurosci.

[43] Loria F, Vargas JY, Bousset L, Syan S, Salles A, Melki R, Zurzolo C (2017). α-Synuclein transfer between neurons and astrocytes indicates that astrocytes play a role in degradation rather than in spreading. Acta Neuropathol.

[44] Rostami J, Holmqvist S, Lindström V, Sigvardson J, Westermark GT, Ingelsson M, Bergström J, Roybon L, Erlandsson A (2017). Human astrocytes transfer aggregated alpha-synuclein via tunneling nanotubes. J Neurosci.

[45] Sofroniew MV, Vinters HV (2010) Astrocytes: biology and pathology. Acta Neuropathol 29 10.1007/s00401-009-0619-8 PMC2799634 20012068

[46] Wilhelmsson U, Bushong EA, Price DL, Smarr BL, Phung V, Terada M, Ellisman MH, Pekny M (2006). Redefining the concept of reactive astrocytes as cells that remain within their unique domains upon reaction to injury. Proc Natl Acad Sci.

[47] Farina C, Aloisi F, Meinl E (2007). Astrocytes are active players in cerebral innate immunity. Trends Immunol.

[48] Lee H-J, Kim C, Lee S-J (2010). Alpha-synuclein stimulation of astrocytes: potential role for neuroinflammation and neuroprotection. Oxid Med Cell Longev.

[49] Zhang W, Wang T, Pei Z, Miller DS, Wu X, Block ML, Wilson B, Zhang W, Zhou Y, Hong J-S, Zhang J (2005). Aggregated α-synuclein activates microglia: a process leading to disease progression in Parkinson’s disease. FASEB J.

[50] Yamanaka K, Chun SJ, Boillee S, Fujimori-Tonou N, Yamashita H, Gutmann DH, Takahashi R, Misawa H, Cleveland DW (2008). Astrocytes as determinants of disease progression in inherited amyotrophic lateral sclerosis. Nat Neurosci.

[51] Chavarria C, Rodríguez-Bottero S, Quijano C, Cassina P, Souza JM (2018). Impact of monomeric, oligomeric and fibrillar alpha-synuclein on astrocyte reactivity and toxicity to neurons. Biochem J.

[52] Chou T-W, Chang NP, Krishnagiri M, Patel AP, Lindman M, Angel JP, Kung P-L, Atkins C, Daniels BP (2021). Fibrillar α-synuclein induces neurotoxic astrocyte activation via RIP kinase signaling and NF-κB. Cell Death Dis.

[53] Fellner L, Irschick R, Schanda K, Reindl M, Klimaschewski L, Poewe W, Wenning GK, Stefanova N (2013). Toll-like receptor 4 is required for α-synuclein dependent activation of microglia and astroglia. Glia.

[54] Klegeris A, Giasson BI, Zhang H, Maguire J, Pelech S, McGeer PL (2006). Alpha-synuclein and its disease-causing mutants induce ICAM-1 and IL-6 in human astrocytes and astrocytoma cells. FASEB J.

[55] Lee E-J, Woo M-S, Moon P-G, Baek M-C, Choi I-Y, Kim W-K, Junn E, Kim H-S (2010). α-Synuclein activates microglia by inducing the expressions of matrix metalloproteinases and the subsequent activation of protease-activated receptor-1. JI.

[56] Reynolds AD, Glanzer JG, Kadiu I, Ricardo-Dukelow M, Chaudhuri A, Ciborowski P, Cerny R, Gelman B, Thomas MP, Mosley RL, Gendelman HE (2008). Nitrated alpha-synuclein-activated microglial profiling for Parkinson’s disease: synuclein-induced microglia activation. J Neurochem.

[57] Reynolds AD, Kadiu I, Garg SK, Glanzer JG, Nordgren T, Ciborowski P, Banerjee R, Gendelman HE (2008). Nitrated alpha-synuclein and microglial neuroregulatory activities. J Neuroimmune Pharmacol.

[58] Reynolds AD, Stone DK, Mosley RL, Gendelman HE (2009). Nitrated α-synuclein-induced alterations in microglial immunity are regulated by CD4+ T cell subsets. J Immunol.

[59] Thomas MP, Chartrand K, Reynolds A, Vitvitsky V, Banerjee R, Gendelman HE (2007). Ion channel blockade attenuates aggregated alpha synuclein induction of microglial reactive oxygen species: relevance for the pathogenesis of Parkinson’s disease. J Neurochem.

[60] Bhattacharjee P, Öhrfelt A, Lashley T, Blennow K, Brinkmalm A, Zetterberg H (2019). Mass spectrometric analysis of Lewy body-enriched α-synuclein in Parkinson’s disease. J Proteome Res.

[61] Kellie JF, Higgs RE, Ryder JW, Major A, Beach TG, Adler CH, Merchant K, Knierman MD (2015). Quantitative measurement of intact alpha-synuclein proteoforms from post-mortem control and Parkinson’s disease brain tissue by intact protein mass spectrometry. Sci Rep.

[62] Moors TE, Maat CA, Niedieker D, Mona D, Petersen D, Timmermans-Huisman E, Kole J, El-Mashtoly SF, Spycher L, Zago W, Barbour R, Mundigl O, Kaluza K, Huber S, Hug MN, Kremer T, Ritter M, Dziadek S, Geurts JJG, Gerwert K, Britschgi M, van de Berg WDJ (2021). The subcellular arrangement of alpha-synuclein proteoforms in the Parkinson’s disease brain as revealed by multicolor STED microscopy. Acta Neuropathol.

[63] Ohrfelt A, Zetterberg H, Andersson K, Persson R, Secic D, Brinkmalm G, Wallin A, Mulugeta E, Francis PT, Vanmechelen E, Aarsland D, Ballard C, Blennow K, Westman-Brinkmalm A (2011). Identification of novel α-synuclein isoforms in human brain tissue by using an online NanoLC-ESI-FTICR-MS method. Neurochem Res.

[64] Killinger BA, Madaj Z, Sikora JW, Rey N, Haas AJ, Vepa Y, Lindqvist D, Chen H, Thomas PM, Brundin P, Brundin L, Labrie V (2018). The vermiform appendix impacts the risk of developing Parkinson’s disease. Sci Transl Med.

