# Supplementary material for: Prominent astrocytic alpha-synuclein pathology with unique post-translational modification signatures unveiled across Lewy body disorders
Source: Acta Neuropathol Commun. 2022 Nov 12;10:163. doi: 10.1186/s40478-022-01468-8 (PMC9652889; doi:10.1186/s40478-022-01468-8)
Supplement: Supplementary file 1 — Additional file 1: Table S1. The primary and secondary antibodies included in this study. AC = autoclave; agg-aSyn = aggregated alpha-synuclein; aSyn = alpha-synuclein; FA = formic acid; GFAP = glial fibrillary acidic protein; Iba1 = ionized calcium binding adaptor protein 1; IF = immunofluorescence; IHC = immunohistochemistry; MBP = myelin basic protein; mc = monoclonal; mus = mouse; na = not applicable; NF = neurofilament; pc = polyclonal; rab = rabbit; SB = slot blot. [file 40478_2022_1468_MOESM1_ESM.pdf]

Suppl. Table 1: The primary and secondary antibodies included in this study.

primary antibodies							
**antibody**	**epitope**	**species/ clonality**	**company / catalogue #**	**IHC: antigen retrieval**	**IHC: dilution**	**IF: dilution***	**SB: dilution**
LASH-EGTNter	aSyn 1-20	rab pc	na	AC+FA	1:15,000	na	1:1,000
LASH-BL 34-45	aSyn 34-45	mus mc	Biolegend #849101	FA	1:30,000	1:10,000	na
LASH-BL 80-96	aSyn 80-96	mus mc	Biolegend #848302	FA	1:20,000	na	na
BD SYN-1	aSyn 91-99	mus mc	BD #BD610787	FA	1:5,000	na	1:2,000
BL 4B12	aSyn 103-108	mus mc	Biolegend #807801	AC+FA	1:100,000	na	na
2F10-E12	aSyn 110-115	mus mc	na	AC+FA	1:10,000	na	na
AB 134-138	aSyn 134-138	rab pc	Abcam #ab131508	AC+FA	1:25,000	na	na
LASH-BL pY39	aSyn pY39	mus mc	Biolegend #849201	AC+FA	1:2,000	1:500	1:1,000
LASH pS87	aSyn pS87	rab pc	na	FA	1:600	na	na
AB pY125	aSyn pY125	rab pc	Abcam #ab10789	FA	1:500	na	na
AB EP1536Y	aSyn pS129	rab mc	Abcam #ab51253	AC+FA	1:60,000	1:10,000	na
AB pY133	aSyn pY133	rab pc	Abcam #ab194910	AC+FA	1:400	na	na
AB pY136	aSyn pY136	rab pc	Abcam #ab131491	FA	1:100	na	na
LASH-EGT nY39	aSyn nY39	rab pc	na	FA	1:1,000	1:500	1:200
6A3-E9	aSyn-120	mus mc	na	AC+FA	1:2,500	na	na
5G4	44-57 // agg-aSyn	mus mc	Merck-Millipore #MABN389	AC+FA	1:5,000	na	na
SYNO4	agg-aSyn	mus mc	na	FA	1:5,000	na	na
Merck-MP anti-GFAP	GFAP	ck pc	Merck-Millipore #AB5541	na	na	1:500	na
Merck-MP EP79 anti-NF	NF	rab mc	Merck-Millipore #302R-1	na	na	1:500	na
AB anti-Iba1	Iba1	goat pc	Abcam #ab5076	na	na	1:80	na
Merck-MP anti-MBP	MBP	rab pc	Merck-Millipore #AB5864	na	na	1:2,000	na
AB EPR8830 recomb anti-Ub	ubiquitin	rab mc	Abcam #ab134953	na	na	1:800	na
Proteintech anti-p62	p62	rab pc	Proteintech #18420-1-AP	na	na	1:200	na
*AC+FA pre-treatment was applied for IF studies for all antibodies.							

secondary antibodies				
**antibody**	**dilution**	**company**	**catalogue #**	**application**
donkey anti-chicken 488	1:400	Jackson ImmunoResearch	703-545-155	IF
goat anti-chicken 568	1:400	ThermoFisher	A-11041	IF
donkey anti-goat 488	1:1,000	ThermoFisher	A-11055	IF
donkey anti-rabbit 568	1:1,000	ThermoFisher	A-10042	IF
donkey anti-mouse 568	1:1,000	ThermoFisher	A-10037	IF
donkey anti-mouse 647	1:1,000	ThermoFisher	A-31571	IF
IRDye goat anti-mouse 680	1:20,000	Li-Cor	926-68070	SB
IRDye goat anti-rabbit 800	1:20,000	Li-Cor	926-32211	SB

AC = autoclave; agg-aSyn = aggregated alpha-synucleina; aSyn = alpha-synuclein; FA = formic acid; GFAP = glial fibrillary acidic protein; Iba1 = ionised calcium binding adaptor protein 1; IF = immunofluorescence; IHC = immunohistochemistry; MBP = myelin basic protein; mc = monoclonal; mus = mouse; na = not applicable; NF = neurofilament; pc = polyclonal; rab = rabbit; SB = slot blot
