# Supplementary material for: Prominent astrocytic alpha-synuclein pathology with unique post-translational modification signatures unveiled across Lewy body disorders
Source: Acta Neuropathol Commun. 2022 Nov 12;10:163. doi: 10.1186/s40478-022-01468-8 (PMC9652889; doi:10.1186/s40478-022-01468-8)

SUPPLEMENTARY FIGURE 1

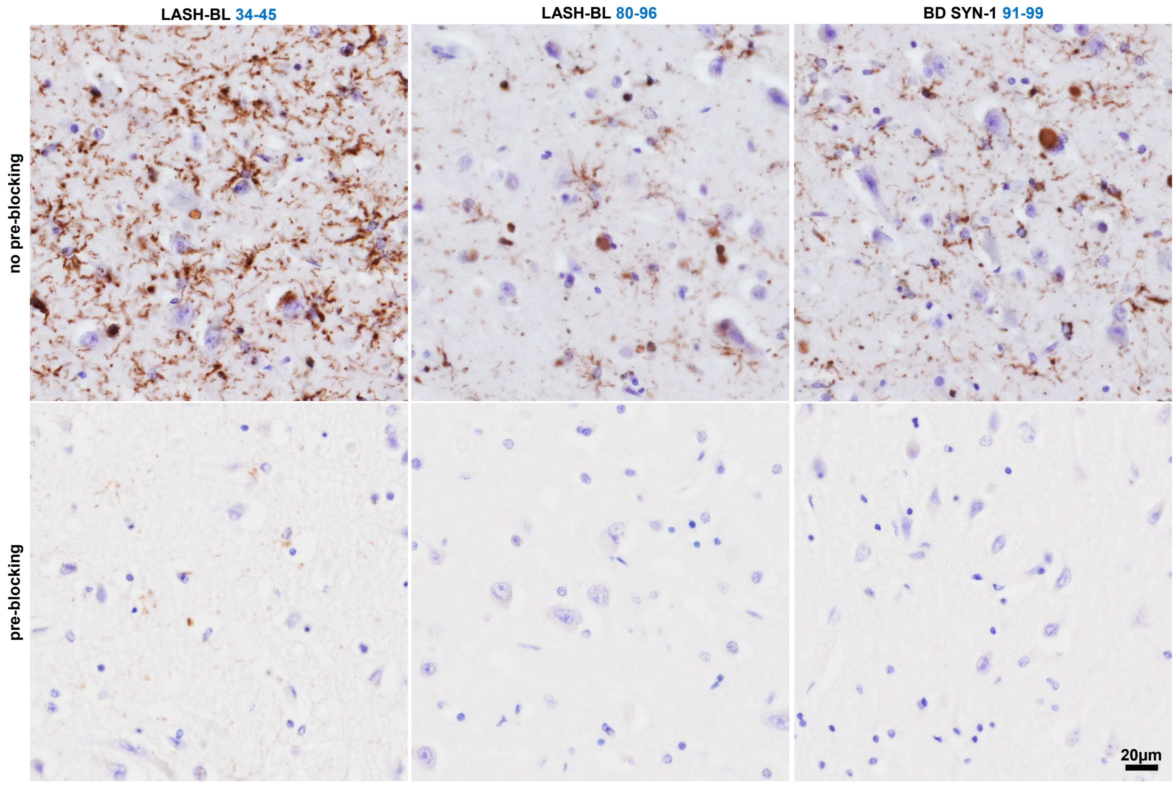

SUPPLEMENTARY FIGURE 2

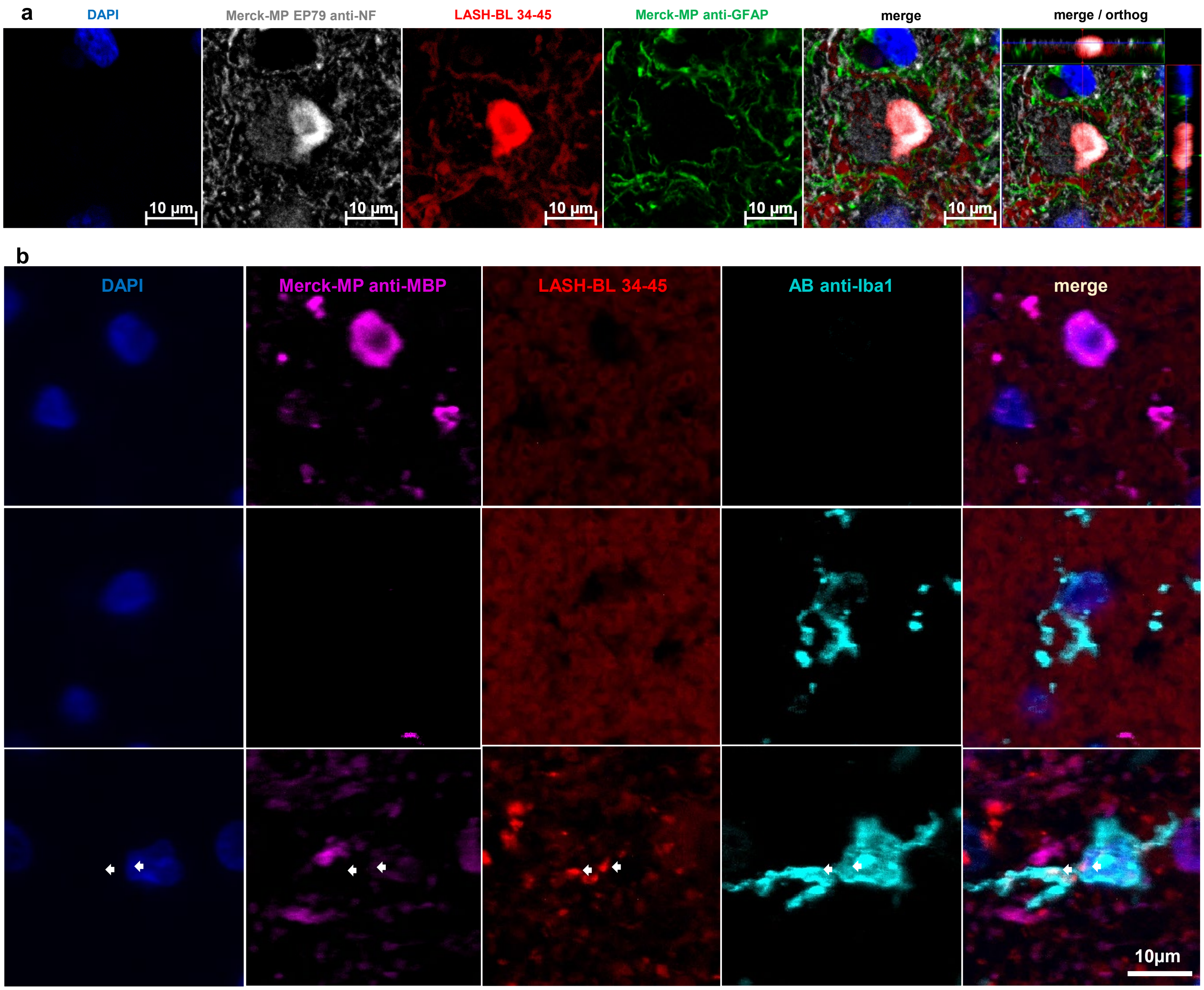

SUPPLEMENTARY FIGURE 3

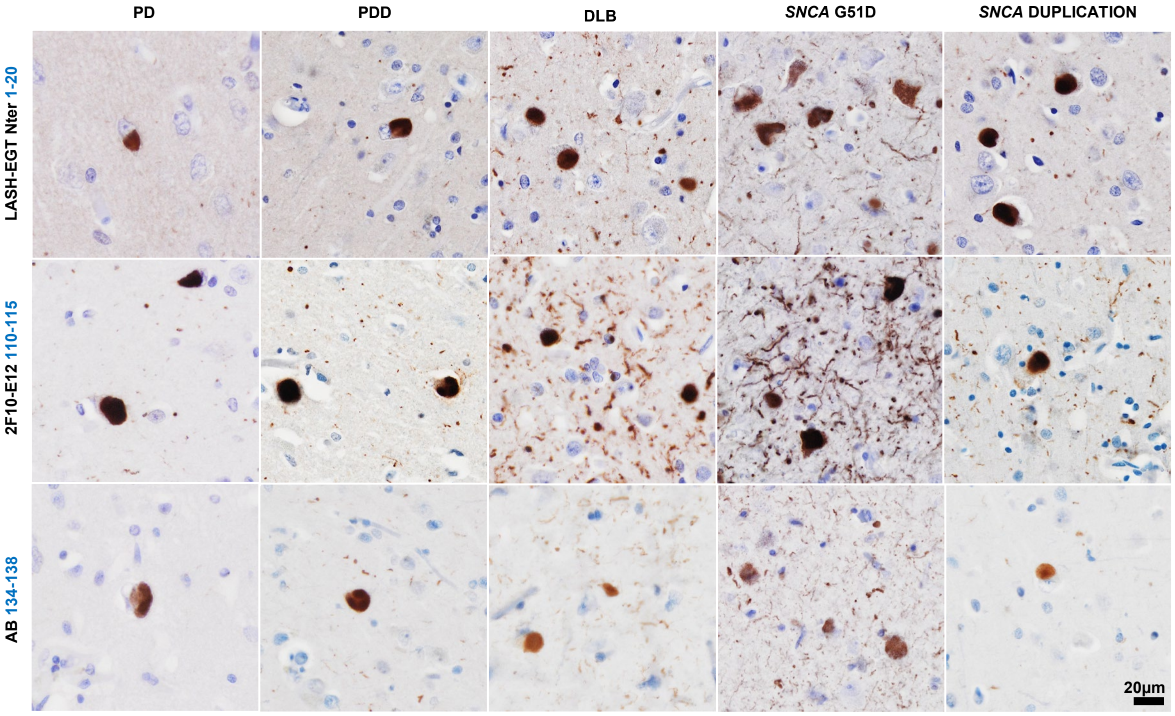

SUPPLEMENTARY FIGURE 4

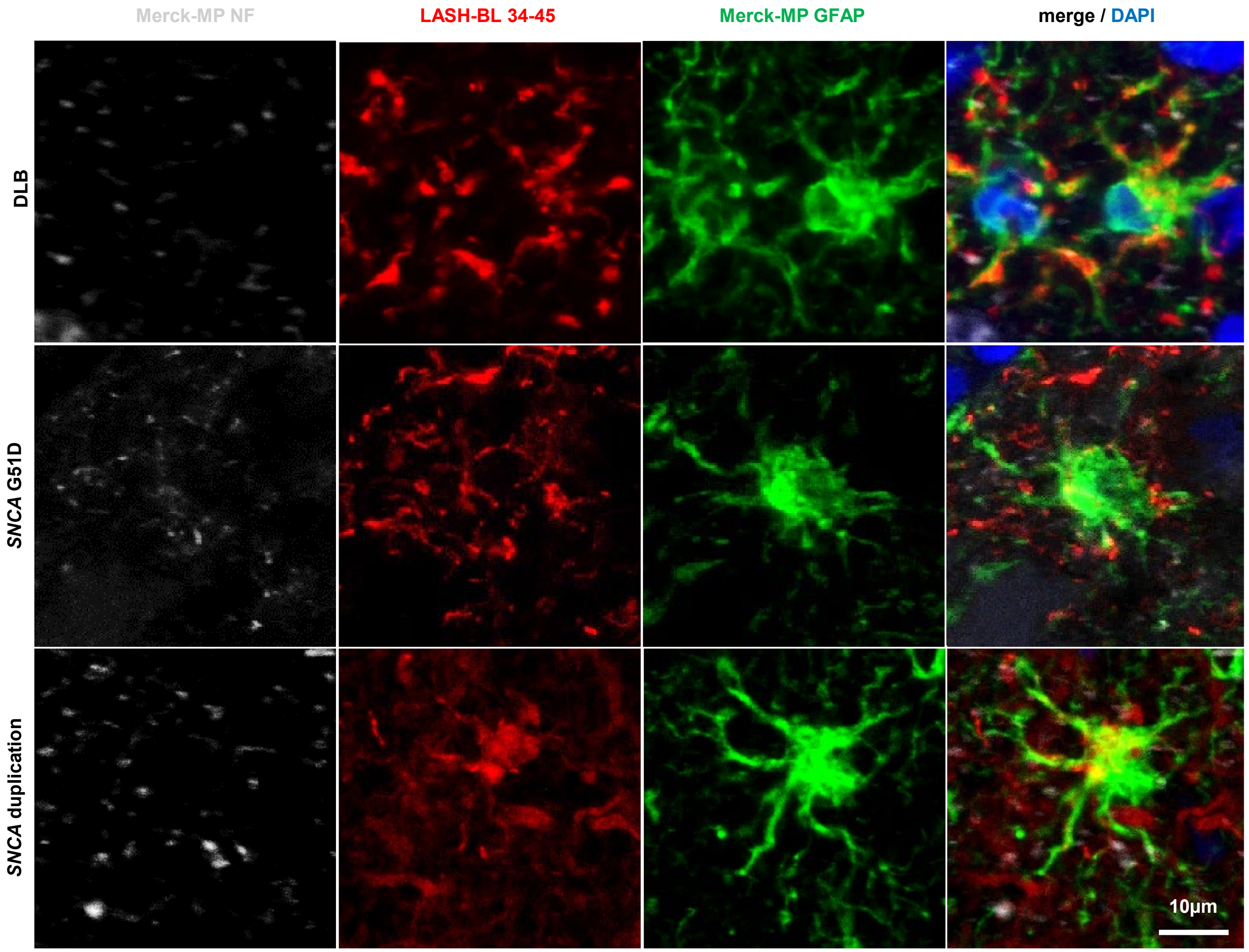

SUPPLEMENTARY FIGURE 5

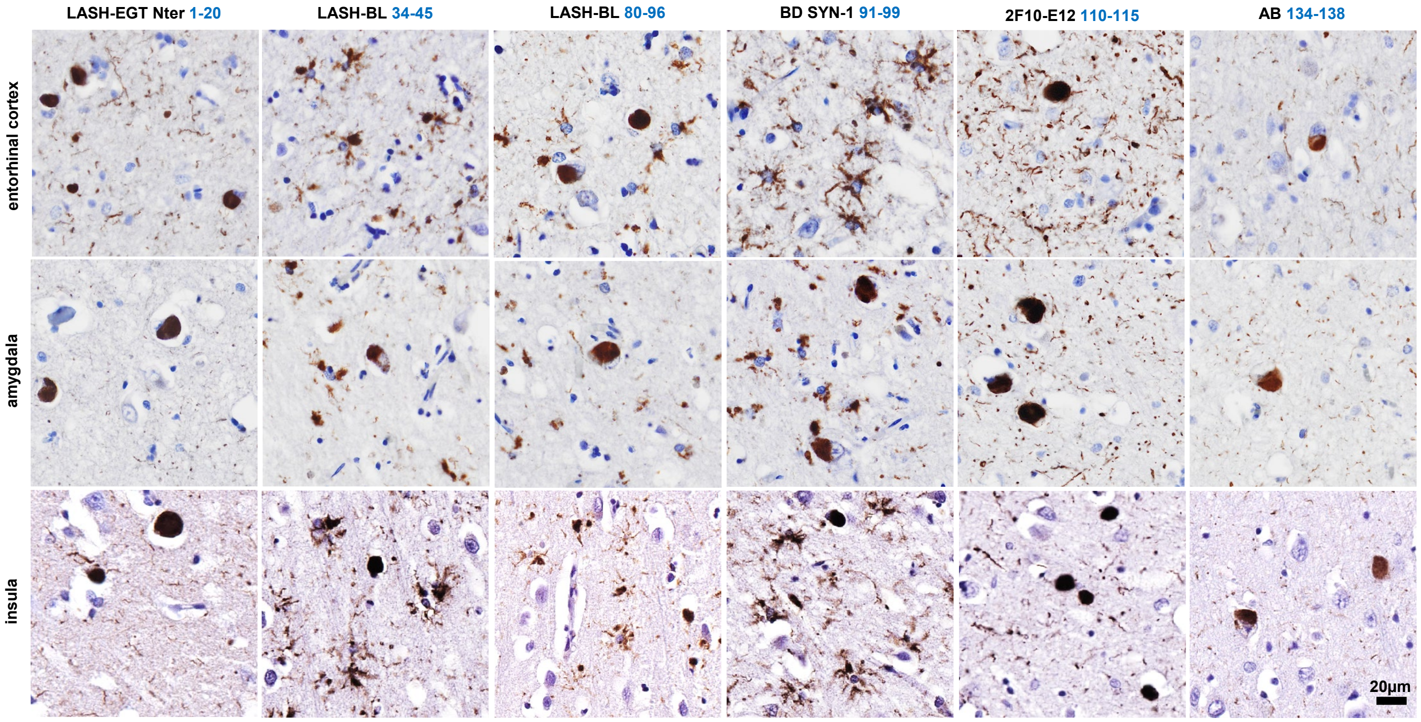

Supplement: Supplementary file 2 — Additional file 2: Fig. S1. Specificity validation of the aSyn antibodies LASH-BL 34-45, LASH-BL 80-96 and BD SYN-1 by pre-adsorption followed by IHC on DLB1 cingulate cortex (layers V–VI). aSyn = alpha-synuclein; DLB = dementia with Lewy bodies; IHC = immunohistochemistry. Fig. S2. (a) A representative image of a cortical LB positive for NF and LASH-BL 34-45, and negative for GFAP. Image from DLB1 cingulate cortex taken using Zeiss LSM700 confocal microscope. (b) The oligodendrocytes and microglial cells, marked by anti-MBP and anti-Iba1 antibodies, respectively, were negative for aSyn in the white matter (upper two panels). Punctate aSyn positivity was detected in the microglial cells in the grey matter (lower panel) as rare events. Images taken from DLB1 cingulate cortex using Olympus slide scanner at 40x magnification. aSyn = alpha-synuclein; DLB = dementia with Lewy body; GFAP = glial fibrillary acidic protein; Iba1 = ionised calcium binding adaptor protein 1; MBP = myelin basic protein; LB = Lewy body; NF = neurofilament. Fig. S3. The cingulate cortex of sporadic PD, PDD, DLB, SNCA G51D mutation and SNCA duplication cases immunostained using antibodies against the N-terminal (LASH-EGTNter) and C-terminal (2F10-E12 and AB 134-138) of aSyn. Astrocytic aSyn was not detected using these antibodies. Representative images taken from cingulate cortical layers V–VI of PD7, PDD5, DLB1, SNCA G51D3 and SNCA duplication cases. aSyn = alpha-synuclein; DLB = dementia with Lewy bodies; PD = Parkinson’s disease; PDD = Parkinson’s disease with dementia. Fig. S4. Representative IF images of GFAP-positive astrocytes from DLB1, SNCA G51D3 and SNCA duplication cingulate cortices, showing positivity for aSyn detected using LASH-BL 34-45 antibody. Images taken using Olympus slide scanner at 40x magnification. aSyn = alpha-synuclein; DLB = dementia with Lewy body; GFAP = glial fibrillary acidic protein; IF = immunofluorescence; NF = neurofilament. Fig. S5. Representative images from [file 40478_2022_1468_MOESM2_ESM.pdf]
